# Supplementary material for: Complementary cortical and striatal encoding of locomotor preparation and performance
Source: iScience. 2025 Nov 19;28(12):114138. doi: 10.1016/j.isci.2025.114138 (PMC12721179; doi:10.1016/j.isci.2025.114138)
Supplement: Document S1. Figures S1–S3 and Table S1 [file mmc1.pdf]

iScience, Volume *28*

Supplemental information

Complementary cortical and striatal encoding of locomotor preparation and performance

Deepak Singla, Long Yang, Andrew S. Weakley, Dylan Davidoff, Jonathan C. Kao, and Sotiris C. Masmanidis

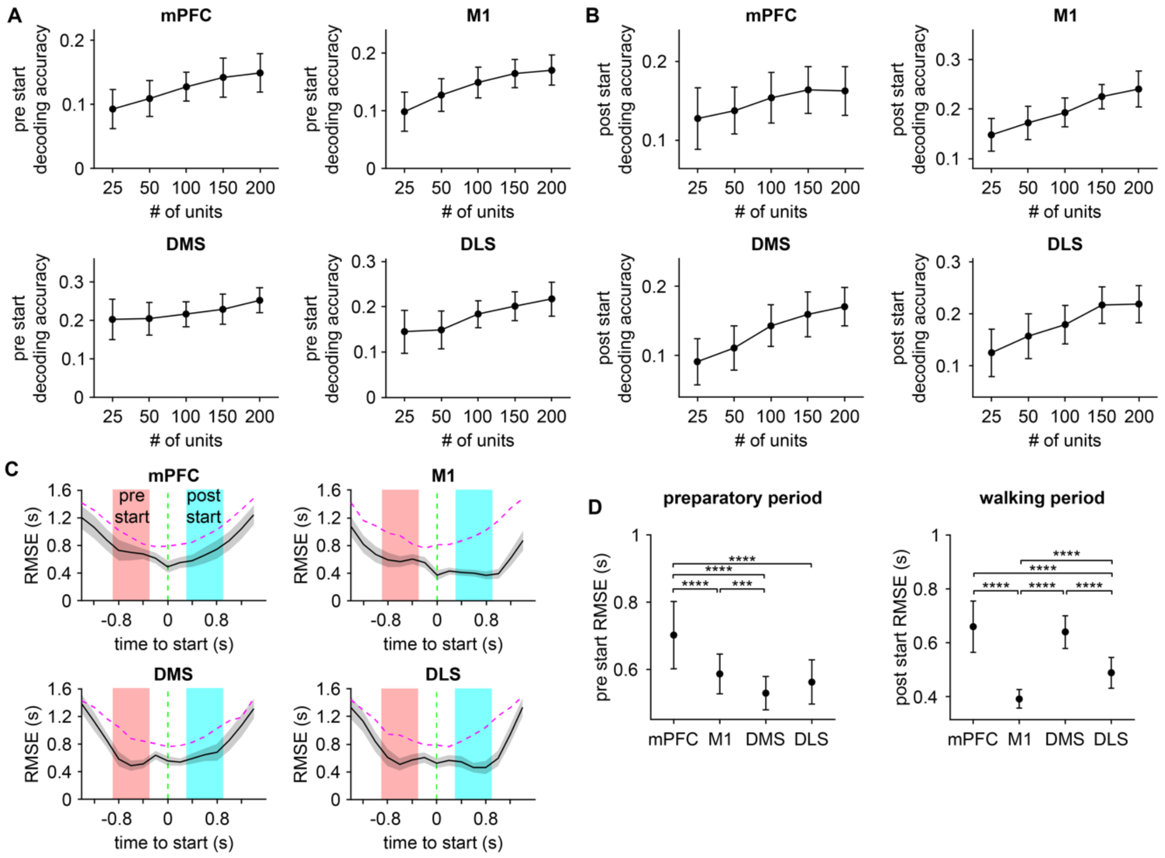

Figure S1. Population decoding of time in the preparatory and performance periods of locomotion, Related to Figure 2.

(A) Mean decoding accuracy in the preparatory ("pre-start") period as a function of the number of neurons. mPFC: Kruskal-Wallis test, $x^2 = 87$, $p < 0.0001$; M1: Kruskal-Wallis test, $x^2 = 115$, $p < 0.0001$; DMS: one-way ANOVA, $F(4,245) = 13$, $p < 0.0001$; DLS: Kruskal-Wallis test, $x^2 = 90$, $p < 0.0001$.

(B) Mean decoding accuracy in the performance ("post-start") period of walking as a function of the number of neurons. mPFC: Kruskal-Wallis test, $x^2 = 39$, $p < 0.0001$; M1: one-way ANOVA, $F(4,245) = 71$, $p < 0.0001$; DMS: Kruskal-Wallis test, $x^2 = 124$, $p < 0.0001$; DLS: Kruskal-Wallis test, $x^2 = 118$, $p < 0.0001$. For **A** and **B**, data represents the mean ± s.d. across 50 random drawings of the specified number of cells.

(C) Mean decoder root mean squared error (RMSE) aligned to the start of walking. Shaded area represents the s.d. across 50 random drawings of 200 cells. Magenta dashed line indicates the 5 % confidence interval of RMSE for time-shuffled data. Green dashed line represents the onset of walking.

(D) Left, mean RMSE in the preparatory period compared across the four areas (one-way ANOVA, $F(3,196) = 56$, $p < 0.0001$). Right, mean RMSE in the performance period of walking compared across the four areas (Kruskal-Wallis test, $x^2 = 153$, $p < 0.0001$). Data represents the mean ± s.d. across 50 random drawings of 200 cells. For the preparatory period, post hoc Tukey-Kramer test was applied for all area-wise comparisons and for the performance period, post hoc Dunn-Sidak test was applied for all area-wise comparisons. ***p<0.001, ****p<0.0001.

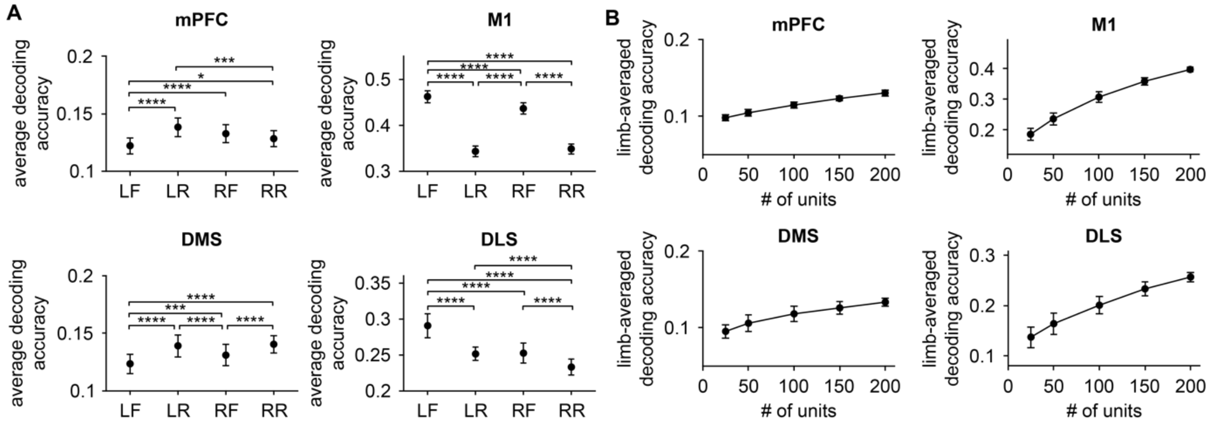

Figure S2. Population decoding of gait phase, Related to Figure 6.

(A) Mean decoding accuracy averaged over the gait cycle compared across the four limbs. mPFC: Friedman test, $\chi^2 = 56$, $p < 0.0001$; M1: one-way RM ANOVA, $F(3,147) = 1397$, $p < 0.0001$; DMS: one-way RM ANOVA, $F(3,147) = 44$, $p < 0.0001$; DLS: one-way RM ANOVA, $F(3,147) = 271$, $p < 0.0001$. Data represents the mean ± s.d. across 50 random drawings of 200 cells. Except for mPFC, post hoc Tukey-Kramer test was applied for all limb-wise comparisons. For mPFC, post hoc Dunn-Sidak test was applied for all limb-wise comparisons.

(B) Mean limb-averaged decoding accuracy as a function of the number of neurons. mPFC: one-way ANOVA, $F(4,245) = 589$, $p < 0.0001$; M1: Kruskal-Wallis test, $\chi^2 = 237$, $p < 0.0001$; DMS Kruskal-Wallis test, $\chi^2 = 178$, $p < 0.0001$; DLS: Kruskal-Wallis test, $\chi^2 = 219$, $p < 0.0001$. Data represents the mean ± s.d. across 50 random drawings of the specified number of cells. *p<0.05, ***p<0.001, ****p<0.0001.

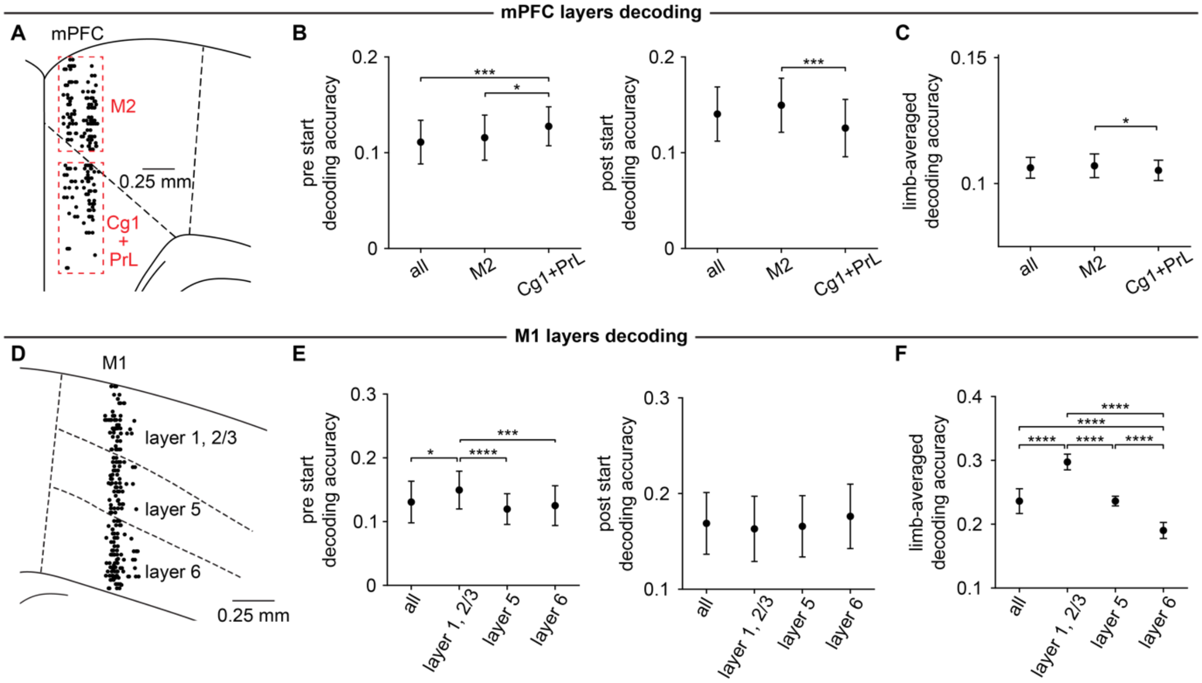

Figure S3. Decoding performance in different mPFC subregions and M1 layers, Related to Figure 2 and Figure 6.

(A) Location of single units recorded from mPFC. The neurons from M2 were selected between DV = 0 mm to 0.75 mm (n = 116 cells pooled), and neurons from Cg1 + PrL were selected between DV = 0.86 mm to 1.67 mm (n = 82 cells pooled).

(B) Left, mean time decoding accuracy in the preparatory ("pre-start", t = -0.9 to -0.3 s) period across the mPFC layers (one-way ANOVA, n = 50 random drawings of 60 cells, F(2,147) = 7.3, p < 0.001). Right, mean time decoding accuracy in the performance ("post-start", t = 0.3 to 0.9 s) period of walking across the mPFC layers (Kruskal-Wallis test, n = 50 random drawings of 60 cells, χ^2 = 16, p < 0.001).

(C) Mean limb-averaged gait decoding accuracy compared across the mPFC layers (Kruskal-Wallis test, n = 50 random drawings of 60 cells, χ^2 = 7.62, p < 0.05). For **B** and **C,** data represents the mean ± s.d. across 50 random drawings of 60 cells. For post start decoding plot in **B**, post hoc Tukey-Kramer test was applied for all layer-wise comparisons. For pre start decoding plot in **B** and **C**, post hoc Dunn-Sidak test was applied for all layer-wise comparisons.

(D) Location of single units recorded from M1. The neurons were divided into 3 cortical layers matching the Allen atlas. Neurons in layer 1, 2/3, DV = 0 mm to 0.4 mm (n = 59 cells pooled); neurons in layer 5, DV = 0.44 mm to 0.78 mm (n = 52 cells pooled); neurons in layer 6, DV = 0.81 mm to 1.24 mm (n = 89 cells pooled).

(E) Left, mean time decoding accuracy in the preparatory ("pre-start", t = -0.9 to -0.3 s) period across the M1 layers (Kruskal-Wallis test, n = 50 random drawings of 50 cells, χ^2 = 32, p < 0.0001). Right, mean time decoding accuracy in the performance ("post-start", t = 0.3 to 0.9 s) period of walking across the M1 layers (one-way ANOVA, n = 50 random drawings of 50 cells, F(3,196) = 1.5, p = 0.23).

(F) Mean limb-averaged gait decoding accuracy compared across the M1 layers (Kruskal-Wallis test, n = 50 random drawings of 50 cells, χ^2 = 165, p < 0.0001). For **E** and **F,** data represents the mean ± s.d. across 50 random drawings of 50 cells. For pre start decoding plot in **E**, post hoc Tukey-Kramer test was applied for all layer-wise comparisons. For post start decoding

plot in **E** and **F**, post hoc Dunn-Sidak test was applied for all layer-wise comparisons. *$p<0.05$, ***$p<0.001$, ****$p<0.0001$.

Area	Mouse #	Age (weeks)	# of sessions	# of cells
mPFC	1	12	4	91
	2	14.7	5	129
M1	1	25.4	2	41
	2	19.8	3	14
	3	16.2	2	11
	4	16.7	1	4
	5	11.8	4	56
	6	13.5	6	40
	7	13.7	3	41
DMS	1	20.2	4	123
	2	17.4	2	9
	3	12.3	3	76
	4	13.9	1	8
DLS	1	11.9	3	64
	2	13.1	2	23
	3	13.6	4	78
	4	15.2	2	49

Table S1: Mice used in the study. Table listing age, number of recording sessions and total number of neurons obtained from each mouse.
